# Hidden aromaticity behind paratropic ring currents in oxoverdazyl and benzotriazinyl radicals

**DOI:** 10.3762/bjoc.22.111

**Published:** 2026-10-07

**Authors:** Rinat F Salikov, Alexander Yu Belyy, Yury V Tomilov

**Affiliations:** 1 N. D. Zelinsky Institute of Organic Chemistry, Russian Academy of Sciences, 47 Leninsky prosp., 119991 Moscow, Russian Federation

**Keywords:** aromatic stabilization energy, aromaticity, Blatter radicals, isodesmic reactions, oxoverdazyl radicals, ring current

## Abstract

Monoradicals occupy a theoretically underdetermined state in aromaticity theory, possessing both aromatic (2*n* + 1) and antiaromatic (2*n*) spin populations. We systematically investigate benzotriazinyl and oxoverdazyl radicals by disentangling their energetic and magnetic aromaticity through isodesmic reactions, NICS calculations, and EDDB analysis. Our results reveal that both systems are energetically aromatic, yet sustain paratropic ring currents, revealing the divergence of energetic and magnetic criteria. EDDB analysis unveils the electronic origin of this divergence: the antiaromatic α-spins with the unpaired electron are largely localized, whereas the aromatic β-spins are extensively delocalized within the heterocyclic rings. This spin-specific localization provides a direct electronic rationale for the observed dichotomy and contributes to the refinement of Mandado's electron rule for odd-electron systems, demonstrating that the two spin populations influence energetic and magnetic properties differently. Oxidation and reduction of the parent radicals lead to convergence of these criteria in closed-shell ions, offering insights into their reversible redox behavior. This study provides a quantitative framework for understanding aromaticity in odd-electron species.

## Introduction

Stable heterocyclic radicals have long fascinated chemists, not only for their intriguing electronic structures but also for their growing utility across materials science, spintronics, spectroscopy and organic synthesis [1–7]. Among these, nitrogen-centered heterocyclic radicals with fully conjugated cyclic systems – such as oxoverdazyls and benzotriazinyls (Blatter radicals) – stand out for their remarkable stability and unique magnetic properties. The stability of these systems is often attributed to extensive spin delocalization over the heterocyclic core [8]. Yet, a fundamental question remains: How do we quantify and reconcile their aromatic character?

The aromaticity of cyclic conjugated radicals presents a fascinating paradox. Mandado [9–10] combined the classical Hückel and Baird rules [11–12] into a single spin-based framework, establishing that aromaticity is achieved when the number of electrons with the same spin equals 2*n +* 1. In Hückel-aromatic singlet species, both α- and β-spin sets contain 2*n +* 1 electrons, whereas in Baird-aromatic triplet species, one spin population holds 2*n +* 1 electrons and the other 2*n +* 3, both odd numbers. This elegant rule, however, leaves monoradicals in a theoretically underdetermined state. A radical with an odd total number of π-electrons necessarily possesses 2*n +* 1 electrons of one spin (an aromatic configuration) and 2*m* of the other (an antiaromatic configuration). This intrinsic duality raises a critical question: Is the aromaticity of such a species defined by its energetic stabilization or by its magnetic response? Previous investigations by Mandado [9–10] and, recently, by Foroutan-Nejad [13] as well as by Proos Vedin et al. [14–15] have suggested that these two criteria can indeed diverge, hinting that radicals, ion-radicals and n,π* excited states occupy a unique crossroads between aromatic and antiaromatic regimes. The divergence between the magnetic properties and other aromaticity criteria was also noted for several other open-shell systems [11,16–18].

**Figure 1:**
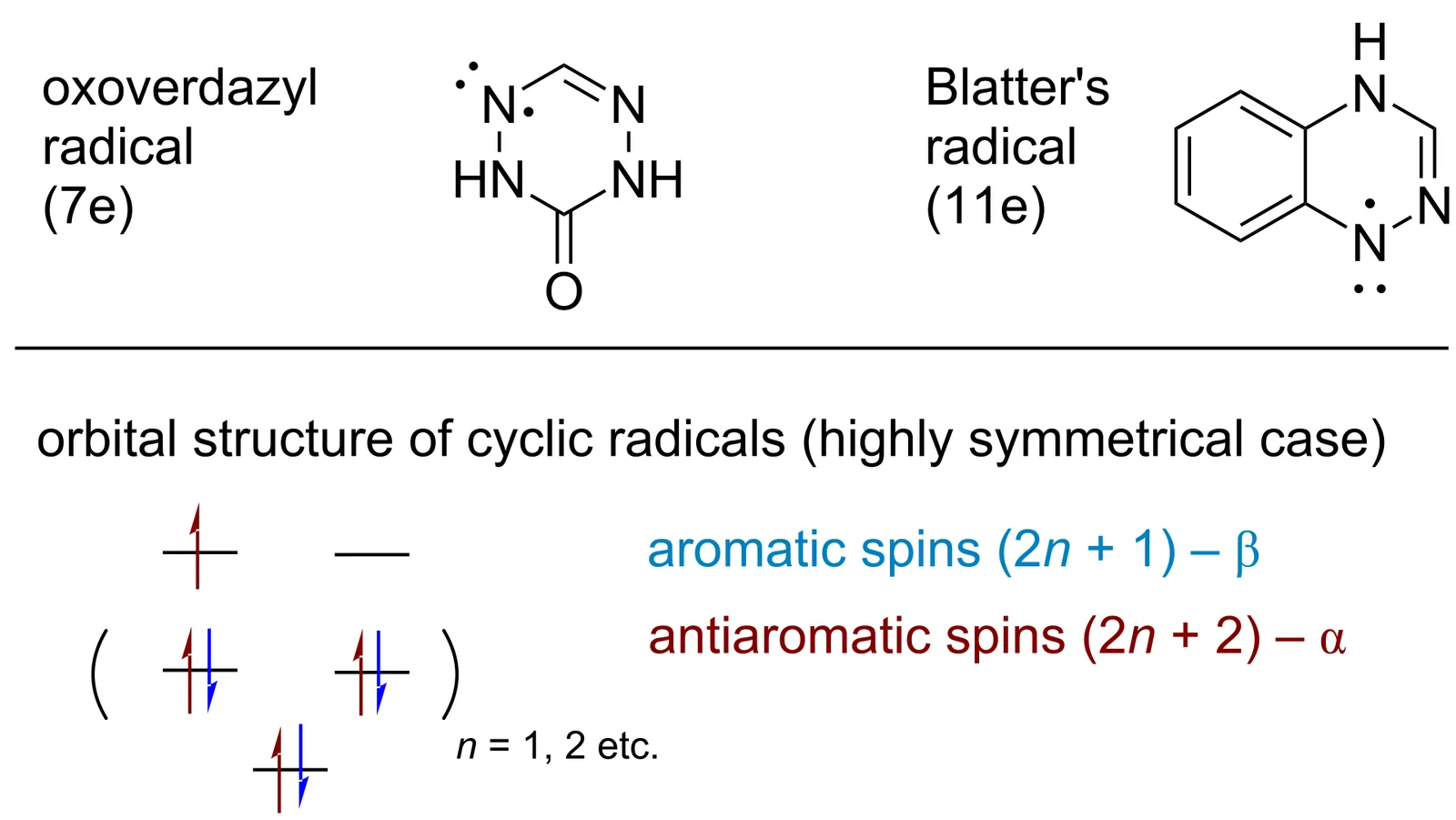
Oxoverdazyl and Blatter radicals (top) and orbital diagram of cyclic-conjugated radicals with 4*n +* 3 π-electrons (bottom).

Our study aims to systematically dissect this dichotomy in the context of oxoverdazyl and benzotriazinyl radicals which possess 2*n +* 1 and 2*n* + 2 electrons of different spins (Figure 1). It is well-established that magnetic indices of antiaromaticity (paratropic ring current) – such as positive nuclear independent chemical shift (NICS) values (the magnetic shielding at a specific point in space using a "ghost atom") [19–24] – often exhibit a significantly larger magnitude than their aromatic counterparts [25]; consequently, the antiaromatic spin component in radicals is expected to dominate the observed magnetic response properties. However, the corresponding energetic consequences, specifically the aromatic stabilization energies (ASEs) arising from both spin populations, remain largely unexplored and form the central focus of our work. By employing the isodesmic reaction approach [26], we will quantify the energetic contribution of cyclic conjugation independently of magnetic criteria, thereby disentangling these two facets of aromaticity and providing a clearer picture of the fundamental forces governing stability in these important radical systems.

## Results and Discussion

**Scheme 1:**
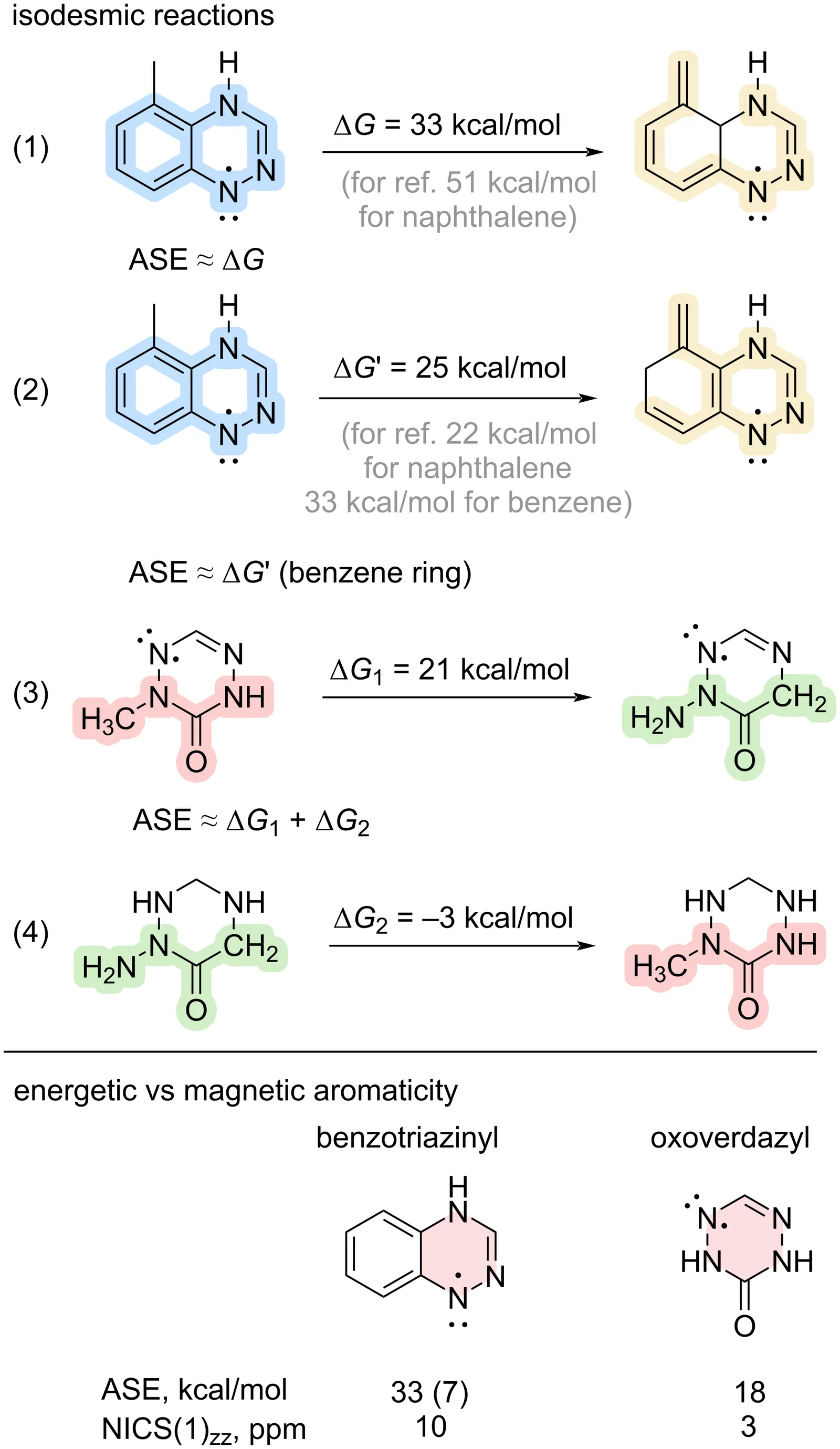
Isodesmic reaction to estimate aromatic stabilization energy of Blatter and oxoverdazyl radicals (top). Energetic vs magnetic criteria of aromaticity of radicals (bottom). Energies were calculated using the dlpno-ccsd(t)/aug-ccTZVP//MP2/def2-TZVP level of theory. NICS(1)_zz_ values were obtained using the PBE0/pcSseg2//MP2/def2-TZVP level of theory.

The aromatic stabilization energy (ASE) of the parent Blatter radical was estimated by calculating the free energy change for the isomerization of its 5-methyl derivative into its regioisomer with interrupted conjugation in both rings (Scheme 1, reaction 1) using a combination of DFT and ab initio post HF methods [27–30] (see Supporting Information File 1). This approach yielded a value of 33 kcal/mol. Interestingly, this value closely matches the ASE of benzene determined by a similar method [31] and smaller than that of naphthalene (51 kcal/mol). To dissect the relative contributions of each ring, we calculated the free energy change for a rearrangement that disrupts conjugation exclusively in the benzene ring, affording a value of 25 kcal/mol. The difference between these two values – 8 kcal/mol – therefore represents the aromatic stabilization specifically attributed to the triazinyl radical ring.

Extending the isodesmic approach to oxoverdazyl is not straightforward, as isomerization would fundamentally alter the conjugated system. We therefore calculated the free energy change for the isomerization of 2-methylverdazyl into its regioisomer with exchanged methylene and amide NH groups (reaction 3). Because this reaction converts a urea fragment into an acyl hydrazide, it would overestimate the ASE. To correct this, we used the reverse reaction with closed-shell hydrogenated analogues lacking cyclic conjugation (reaction 4). The sum of the free energy changes from both reactions gave an ASE of 18 kcal/mol for the oxoverdazyl radical – approximately half that of benzene. Both values obtained are consistent with the near-planar geometry of the heterocyclic rings, unlike the puckered 8-electron 1,4-thiazines [32].

**Figure 2:**
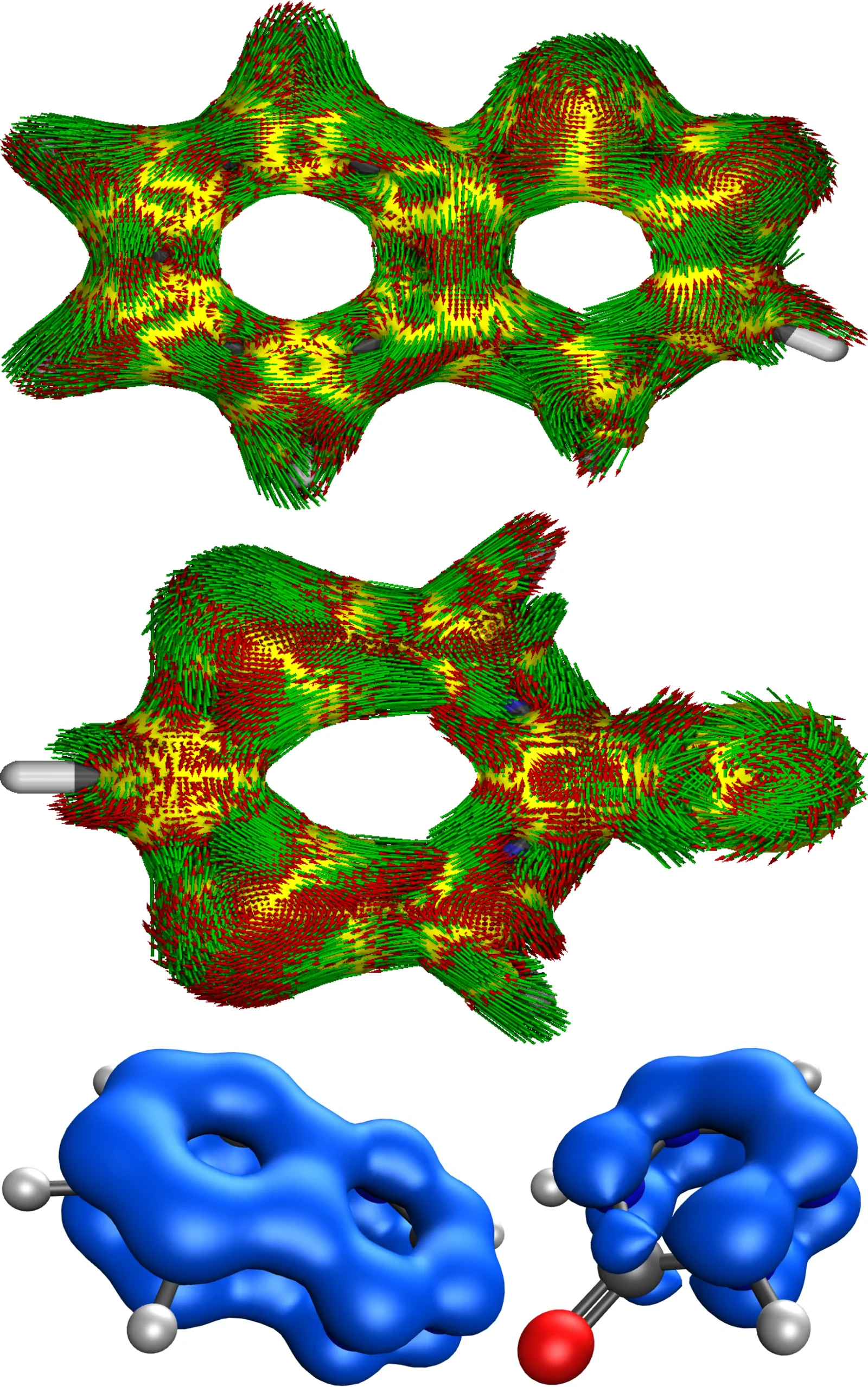
Anisotropy of the induced current density (ACID) diagrams of verdazyl and benzotriazinyl radicals (left) with the directions of ring currents in each ring (the magnetic field is directed towards the viewer). Visualization of delocalized electron density populations with the EDDB method (right); the plot for the oxoverdazyl radical was generated with a tracking pathway.

The magnetic aromatic properties of several oxoverdazyl radicals have been investigated using the gauge-including magnetically induced current-density (GIMIC) method [33], revealing a weakly antiaromatic character. More recently, NICS(1)_zz_ values were calculated for a series of Blatter radicals, indicating a paratropic (antiaromatic) ring current in the triazinyl ring and a diatropic (aromatic) ring current in the benzene ring [34]. To complete this picture, we calculated NICS(1)_zz_ values for the heterocyclic rings of both parent radicals and visualized the ring currents using anisotropy of the induced current density (ACID) diagrams [35] (Scheme 1, Figure 2). Our calculations confirm the presence of weak paratropic ring currents in both heterocyclic cores. These magnetic signatures, however, contrast with the energetic aromaticity quantified in the previous section, underscoring the dichotomy between magnetic and energetic criteria in these systems.

Previous studies [9–10,13,33,36–38] have revealed that the magnetic response of odd-electron systems is often dominated by the stronger paratropic component arising from the antiaromatic α-spin populations (2*n*). To elucidate the electronic origin of the divergence between energetic and magnetic aromaticity, we performed electron density of delocalized bonds (EDDB) analysis [40], which isolates and plots the spatial distribution of delocalized electrons (Table 1, Figure 2). The Blatter radical, owing to the fused benzene ring, demonstrated robust delocalization for both spin populations – 60% of α-electrons (3.6 of 6) and 76% of β-electrons (3.8 of 5), relative to 89% in benzene. However, pathway tracking through the heterocyclic rings, excluding the benzene ring and the carbonyl group, revealed a striking disparity between the two spin sets in both radicals. Notably, the electron density (ED) in the heterocyclic ring in benzotriazinyl corresponds to 6.4 (3.4 α and 3.0 β) electrons (not 7) as it is shifted to the benzene ring. The antiaromatic α-spins were found to be largely localized, with delocalized populations of only 10–18% (0.3 of 3.4 in Blatter’s radical and 0.7 of 4 in oxoverdazyl), whereas the aromatic β-spins were significantly more delocalized, reaching 57–60% (1.7 of 3.0 in Blatter’s radical and 1.8 of 3 in oxoverdazyl). Our EDDB analysis reveals that the stability of these radicals has been mistakenly attributed to delocalization of the unpaired electron. In fact, the dominant contribution to thermodynamic stabilization comes from the delocalization of the electrons with the opposite spin – the aromatic β-spins – within the heterocyclic ring.

**Table 1:** EDDB analysis in benzotriazinyl and oxoverdazyl radicals.

	Benzotriazinyl	Oxoverdazyl

ED	11 (6.4)*	7
ED (α)	6 (3.4)*	4
ED (β)	5 (3.0)*	3
EDDB	7.4 (2.09)*	2.56
EDDB (α)	3.6 (0.3)*	0.7
EDDB (β)	3.8 (1.7)*	1.8

*Values in parentheses correspond to the heterocyclic ring in benzotriazinyl.

**Scheme 2:**
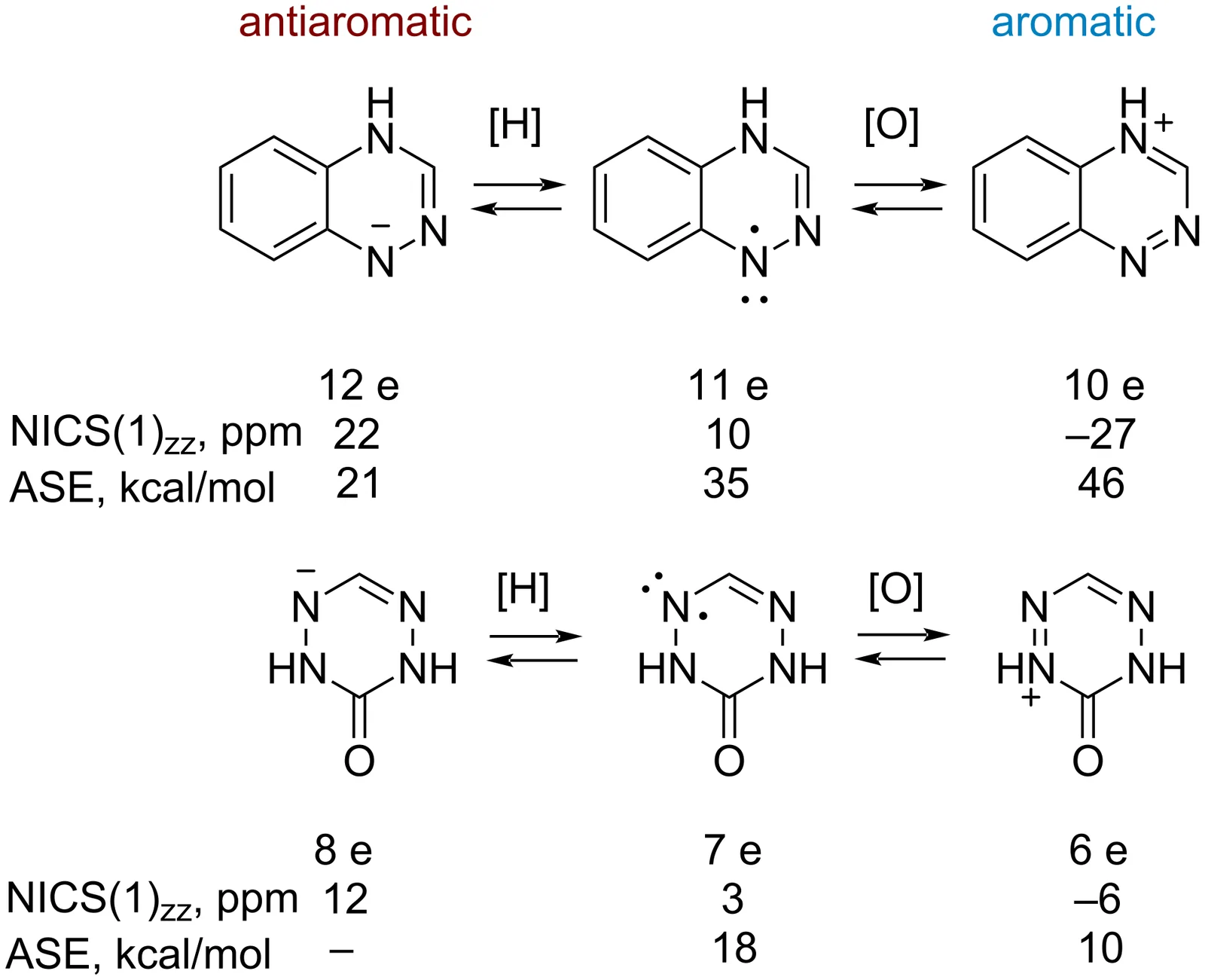
Oxidation and reduction of oxoverdazyl and Blatter radicals and aromatic indices of corresponding cations and anions.

Both classes of radicals are known for their reversed oxidation and reduction processes as evidenced from cyclic voltammetry data [40–41]. Notably, oxidation gives 4*n + 2* electron cations, whereas reduction affords 4*n*-electron anions. To explore the aromatic character of these redox products, we calculated NICS(1)_zz_ values and ASEs for the cations and anions derived from the parent radicals, using the same isodesmic approach employed for the neutral species. Oxidation of the Blatter radical yields a strongly negative NICS(1)_zz_ value in the heterocyclic ring – characteristic of aromatic systems – accompanied by an increase in ASE to 46 kcal/mol, a value close to that of naphthalene (≈50 kcal/mol) [31]. Conversely, reduction amplifies the positive NICS(1)_zz_ value and decreases the ASE to 21 kcal/mol, indicating a slight destabilization induced by the formation of an 8-electron ring system (Scheme 2). The oxoverdazyl radical exhibited a similar magnetic response upon redox transformation. Oxidation led to a significant decrease in the ASE value showing a unique case with stronger stabilization in a seven-electron state relative to that in the six-electron cation. However, the lower value could also be underestimated. For the reduced anion, estimation of an antiaromatic destabilization energy even proved inadequate (see Supporting Information File 1), as the negative charge cannot be effectively delocalized in any suitable reference molecule.

## Conclusion

Through this investigation, we have determined that the two classes of nitrogen-heterocyclic radicals – benzotriazinyl and oxoverdazyl – are best described as energetically aromatic systems that happen to sustain a paratropic (antiaromatic) ring current – a manifestation of α/β conflicting aromaticity. The hidden energetic aromaticity is directly responsible for the planarity of the heterocyclic rings and the remarkable stability observed in both radical classes. Applying an isodesmic approach, we have quantified the aromatic stabilization energies of these systems and established that their magnetic signatures alone do not reflect their true thermodynamic stability.

The mechanistic basis for this dichotomy was uncovered through analysis of electron density of delocalized bonds (EDDB). By tracking delocalization pathways through the heterocyclic rings, we found that the antiaromatic α-spins, including the unpaired electron, are predominantly localized, whereas the aromatic β-spins exhibit extensive delocalization. This asymmetry confirms that the β-spins are primarily responsible for the energetic stabilization, while the α-spins govern the magnetic response – a finding that directly rationalizes the divergence between the two aromaticity criteria. Ultimately, our study offers a contribution to the interpretation of Mandado's spin-separated rule for aromaticity in open-shell systems.

Additionally, our analysis of the redox products reveals how aromatic indices reconverge in closed-shell ions upon oxidation and reduction, offering a deeper understanding of the reversible electrochemistry that characterizes these radicals.

## Computational Methods

Quantum chemical calculations were performed using ORCA 5.0.4 [42–43]. Initial geometries were optimized and frequencies were calculated at the r^2^SCAN-3c level of theory [30], verifying minima with zero imaginary frequencies [31]. Next, the geometries were improved at the MP2/def2-TZVP level of theory, single-point energies were obtained at the DLPNO-CCSD(T)/aug-cc-pVTZ level of theory [27–28]. At the MP2 level of theory, the RIJCOSX approximation was used together with the auxiliary basis sets def2/J and def2-TZVP/C. At the DLPNO-CCSD(T) level of theory, the RIJK approximation was used together with the auxiliary basis sets aug-cc-pVTZ/C and aug-cc-pVTZ/JK, and the TightSCF keyword was applied. In both cases, the FROZENCORE approximation, which is enabled by default in ORCA 5.0.4, was employed.

NICS values were calculated at the PBE0(D4)/pcsSeg-2// MP2/def2-TZVP level of theory. ACID diagrams were obtained at the B3LYP/6-31G(d)//MP2/def2-TZVP level of theory and visualized in AICD-3.0.4 program [36]. The magnetic field is directed towards the viewer, POV is placed on the Z-axis. Electron Density of Delocalized Bonds (EDDB) calculations were performed with runEDDB [39] (https://aromaticity.uj.edu.pl/runeddb/). The electron density was calculated in ORCA 6.1.0, at the B3LYP/def2-TZVP theory level.

## Supporting Information

File 1 Quantum chemical calculation details.

File 2 Anisotropy of the induced current density (ACID) diagrams.

File 3 xyz-Files.

## Data Availability

All data that supports the findings of this study is available in the published article and/or the supporting information of this article.
