# Supplementary material for: Hidden aromaticity behind paratropic ring currents in oxoverdazyl and benzotriazinyl radicals
Source: Beilstein J Org Chem. 2026 Oct 7;22:1376–81. doi: 10.3762/bjoc.22.111 (PMC13656023; doi:10.3762/bjoc.22.111)
Supplement: Supplementary file 1 [file Beilstein_J_Org_Chem-22-1376-s001.pdf]

BEILSTEIN JOURNAL OF ORGANIC CHEMISTRY

Supporting Information

for

Hidden aromaticity behind paratropic ring currents in oxoverdazyl and benzotriazinyl radicals

Rinat F. Salikov, Alexander Yu. Belyy and Yury V. Tomilov

Beilstein J. Org. Chem. **2026,** *22,* 1376–1381. doi:10.3762/bjoc.22.111

Quantum chemical calculation details

License and Terms: This is a supporting information file under the terms of the Creative Commons Attribution License (https://creativecommons.org/licenses/by/4.0). Please note that the reuse, redistribution and reproduction in particular requires that the author(s) and source are credited and that individual graphics may be subject to special legal provisions.
The license is subject to the *Beilstein Journal of Organic Chemistry* terms and conditions: (https://www.beilstein-journals.org/bjoc/terms)

Table of contents

1. Computational data.. S2
2. References.. S6

1. Computational data

Quantum chemical calculations were performed using ORCA 5.0.4 [1,2]. Initial geometries were optimized and frequencies were calculated at the r²SCAN-3c level of theory [3], verifying minima with zero imaginary frequencies [4]. Next, the geometries were improved at the MP2/def2-TZVP level of theory, single-point energies were obtained at the DLPNO-CCSD(T)/aug-cc-pVTZ level of theory [5,6]. At the MP2 level of theory, the RIJCOSX approximation was used together with the auxiliary basis sets def2/J and def2-TZVP/C. At the DLPNO-CCSD(T) level of theory, the RIJK approximation was used together with the auxiliary basis sets aug-cc-pVTZ/C and aug-cc-pVTZ/JK, and the TightSCF keyword was applied. In both cases, the FROZENCORE approximation, which is enabled by default in ORCA 5.0.4, was employed.
In all cases where energetic parameters were considered, the following expression for Gibbs free energy of formation was used:

$$\Delta G_f(X) = \Delta G_f(\mathrm{r2SCAN-3c}) - E_{SPE}(\mathrm{r2SCAN-3c}) + E_{SPE}(\mathrm{DLPNO-CCSD(T)/aug-ccpVTZ}).$$

NICS values were calculated at the PBE0(D4)/pcsSeg-2// MP2/def2-TZVP level of theory. ACID diagrams were obtained at the B3LYP/6-31G(d)//MP2/def2-TZVP level of theory and visualized in AICD-3.0.4 program [7]. Magnetic field is directed towards the viewer, POV is placed on the Z-axis. Electron Density of Delocalized Bonds (EDDB) calculations were performed with runEDDB [8] (https://aromaticity.uj.edu.pl/runeddb/). The electron density was calculated in ORCA 6.1.0, at the B3LYP/def2-TZVP theory level.

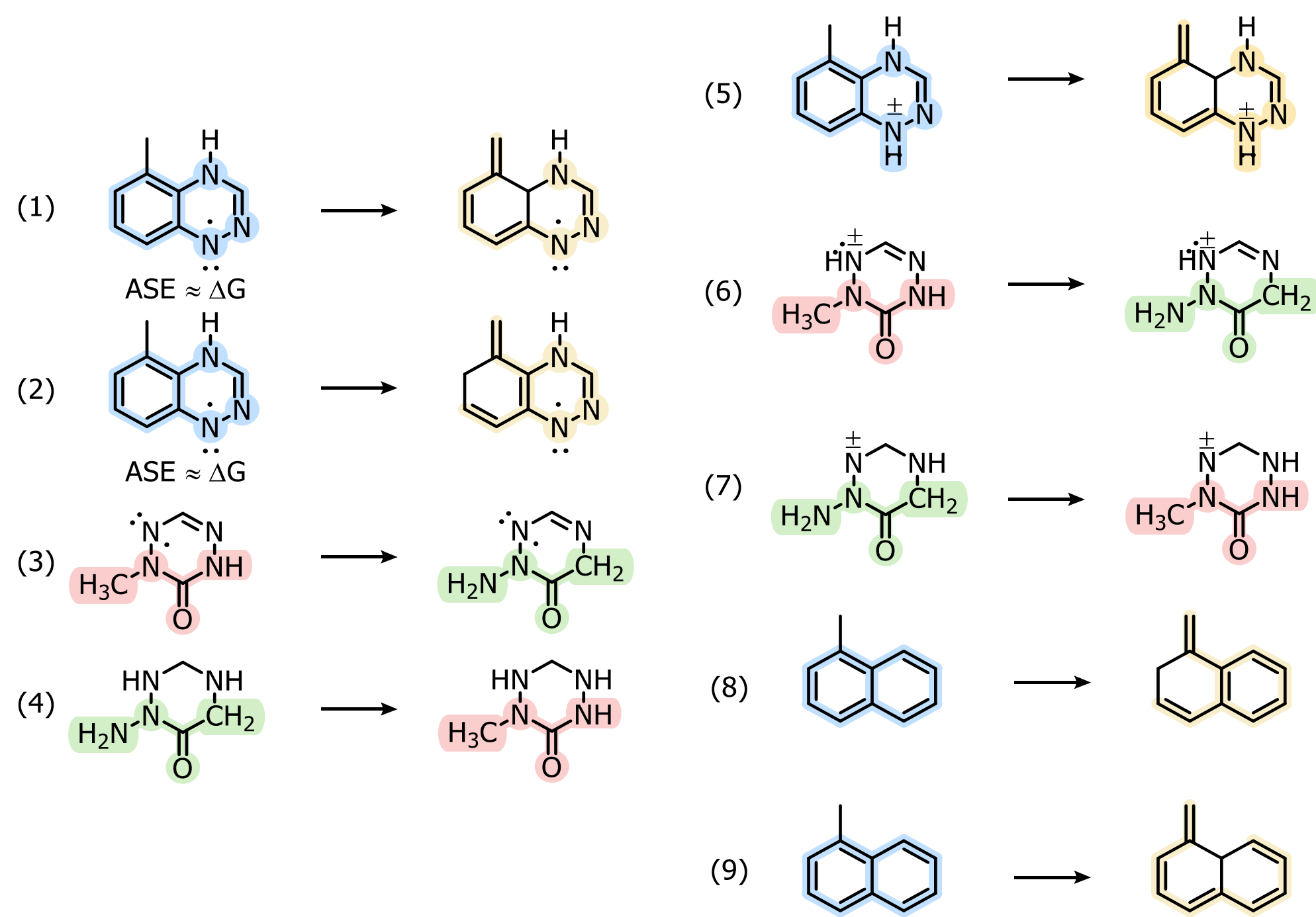

Scheme S1: Isodecmic reaction equations.

Table S1. Energies of optimized structures (isodesmic reactions) with the number of imaginary frequencies. Calculated at DLPNO-CCSD(T)/aug-ccpVTZ// MP2/def2-TZVP level of teory.

compound	Number of imaginary frequencies.	SPE E_h	G E_h	H E_h
**benzotriazinyl_anion_parent.xyz**	0	-433.8457978	-433.7542291	-433.7141444
**benzotriazinyl_cation_parent.xyz**	0	-433.5693641	-433.47184	-433.432739
**benzotriazinyl_parent.xyz**	0	-433.8021352	-433.7086096	-433.6680698
**eq1_eq2_reactant.xyz**	0	-473.0488915	-472.9293368	-472.885224
**eq1_product.xyz**	0	-472.9955681	-472.8770696	-472.8331284
**eq2_product.xyz**	0	-473.0064105	-472.8888758	-472.8437889
**eq3_product.xyz**	0	-410.8350878	-410.7539498	-410.7142978
**eq3_reactant.xyz**	0	-410.8692523	-410.7875564	-410.748856
**eq4_product.xyz**	0	-412.6863575	-412.5614497	-412.521265
**eq4_reactant.xyz**	0	-412.6807036	-412.5572988	-412.5170149
**eq5_anion_product.xyz**	0	-473.0591678	-472.9407558	-472.8981795
**eq5_anion_reactant.xyz**	0	-473.0918236	-472.9741825	-472.9306
**eq5_cation_product.xyz**	0	-472.7468868	-472.6243427	-472.5818108
**eq5_cation_reactant.xyz**	0	-472.8205712	-472.697143	-472.6543904
**eq6_anion_product.xyz**	0	-410.915033	-410.8339454	-410.7946585
**eq6_anion_reactant.xyz**	0	-410.9210591	-410.8405121	-410.8006009
**eq6_cation_product.xyz**	0	-410.5642363	-410.4840085	-410.444465
**eq6_cation_reactant.xyz**	0	-410.5914093	-410.5080623	-410.469535
**eq7_anion_product.xyz**	0	-412.6424202	-412.5219374	-412.4818318
**eq7_anion_reactant.xyz**	0	-412.65275	-412.5327427	-412.4925899
**eq7_cation_product.xyz**	0	-412.4046048	-412.2834923	-412.2410415
**eq7_cation_reactant.xyz**	0	-412.3964376	-412.2747817	-412.2336705
**verdazinium_parent.xyz**	0	-371.3360443	-371.2801644	-371.2448856
**verdazyde_parent.xyz**	0	-371.6832422	-371.6302155	-371.593841
**verdazyl_parent.xyz**	0	-371.6300276	-371.5763512	-371.5398186
**eq8_eq9_reactant_amenapht**	0	-424,4220283	-424,2837485	-424,241332
**eq8_product _iso1amenapht**	0	-424,385587	-424,2484788	-424,2060576
**eq9_product_iso2amenapht**	0	-424,3386739	-424,2028023	-424,1603729

Table S2. Set of calculated NICS values for compounds. Calculated at PBE0(D4)/pcsSeg-2// MP2/def2-TZVP level of theory.

compound	nics(0)	Nics(1)	nics(1)$_{zz}$
**benzotriazinyl_anion_parent.xyz**	11.3	6.2	21.8
**benzotriazinyl_cation_parent.xyz**	-5.7	-10.3	-26.7
**benzotriazinyl_parent.xyz**	8.3	2.7	10.2
**eq1_eq2_reactant.xyz**	8.4	2.7	10.5
**eq1_product.xyz**	-1.4	-3.4	-4.8
**eq3_product.xyz**	4.6	0.6	3.7
**eq3_reactant.xyz**	3.5	0.5	3.3
**eq4_product.xyz**	-2.6	-1.0	2.0
**eq4_reactant.xyz**	-0.8	-0.8	3.0
**verdazinium_parent.xyz**	2.7	-3.1	-6.4
**verdazyde_parent.xyz**	4.0	3.6	12.3
**verdazyl_parent.xyz**	3.5	0.8	3.2

Table S3. EDDB analysis of parent benzotriazinyl radical.

	Total	per atom
ED_G population:	10.95	0.68
- alpha spin:	5.97	0.37
- beta spin:	4.98	0.31
EDLB_G population:	3.58	0.22
- alpha spin:	2.41	0.15
- beta spin:	1.16	0.07
EDDB_G population:	7.37	0.46
- alpha spin:	3.56	0.22
- beta spin:	3.82	0.24

Table S4. EDDB analysis of parent benzotriazinyl radical with tracking through the heterocyclicl ring.

	Total		per atom:
ED_P population:	6.48		1.08
- alpha spin:	3.44		0.57
- beta spin:	3.04		0.51
EDLB_P population:	4.39		0.73
- alpha spin:	3.1		0.52
- beta spin:	1.29		0.22
EDDB_P population:	2.09		0.35
- alpha spin:	0.34		0.06
- beta spin:	1.74		0.29
Cyclic Delocal. Index (CDI):		0.22	
- alpha spin:		0.03	
- beta spin:		0.16	

Table S5. EDDB analysis of parent oxoverdazyl radical with tracking through the heterocyclicl ring.

	Total		per atom
ED_P population:	6.95		1.16
- alpha spin:	3.97		0.66
- beta spin:	2.98		0.5
EDLB_P population:	4.4		0.73
- alpha spin:	3.23		0.54
- beta spin:	1.17		0.19
EDDB_P population:	2.56		0.43
- alpha spin:	0.74		0.12
- beta spin:	1.82		0.3
Cyclic Delocal. Index (CDI):		0.13	
- alpha spin:		0.03	
- beta spin:		0.1	

2. References

(1) Neese, F. *WIREs Comput. Mol. Sci.* **2022**, *12*, e1606. doi:10.1002/wcms.1606

(2) Neese, F. *WIREs Comput. Mol. Sci.* **2012**, *2*, 73–78. doi:10.1002/wcms.81

(3) Grimme, S.; Hansen, A.; Ehlert, S.; Mewes, J.-M. *J. Chem. Phys.* **2021**, *154*, 064103. doi:10.1063/5.0040021

(4) Kesharwani, M. K.; Brauer, B.; Martin, J. M. L. *J. Phys. Chem. A* **2015**, *119*, 1701–1714. doi:10.1021/jp508422u

(5) Riplinger, C.; Sandhoefer, B.; Hansen, A.; Neese, F. *J. Chem. Phys.* **2013**, *139*, 134101. doi:10.1063/1.4821834

(6) Liakos, D. G.; Guo, Y.; Neese, F. *J. Phys. Chem. A* **2020**, *124*, 90–100. doi:10.1021/acs.jpca.9b05734

(7) Geuenich, D.; Hess, K.; Köhler, F.; Herges, R. *Chem. Rev.* **2005**, *105*, 3758–3772. doi:10.1021/cr0300901

(8) Szczepanik, D. W.; Andrzejak, M.; Dominikowska, J.; Pawełek, B.; Krygowski, T. M.; Szatylowicz, H.; Solà, M. *Phys. Chem. Chem. Phys.* **2017**, *19*, 28970–28981. doi:10.1039/C7CP06114E
