# Supplementary material for: Hidden aromaticity behind paratropic ring currents in oxoverdazyl and benzotriazinyl radicals
Source: Beilstein J Org Chem. 2026 Oct 7;22:1376–81. doi: 10.3762/bjoc.22.111 (PMC13656023; doi:10.3762/bjoc.22.111)

BEILSTEIN JOURNAL OF ORGANIC CHEMISTRY

Supporting Information

for

Hidden aromaticity behind paratropic ring currents in oxoverdazyl and benzotriazinyl radicals

Rinat F. Salikov, Alexander Yu. Belyy and Yury V. Tomilov

Beilstein J. Org. Chem. **2026,** *22,* 1376–1381. doi:10.3762/bjoc.22.111

Anisotropy of the induced current density (ACID) diagrams

License and Terms: This is a supporting information file under the terms of the Creative Commons Attribution License (https://creativecommons.org/licenses/by/4.0). Please note that the reuse, redistribution and reproduction in particular requires that the author(s) and source are credited and that individual graphics may be subject to special legal provisions.
The license is subject to the *Beilstein Journal of Organic Chemistry* terms and conditions: (https://www.beilstein-journals.org/bjoc/terms)

benzotriazinyl anion parent

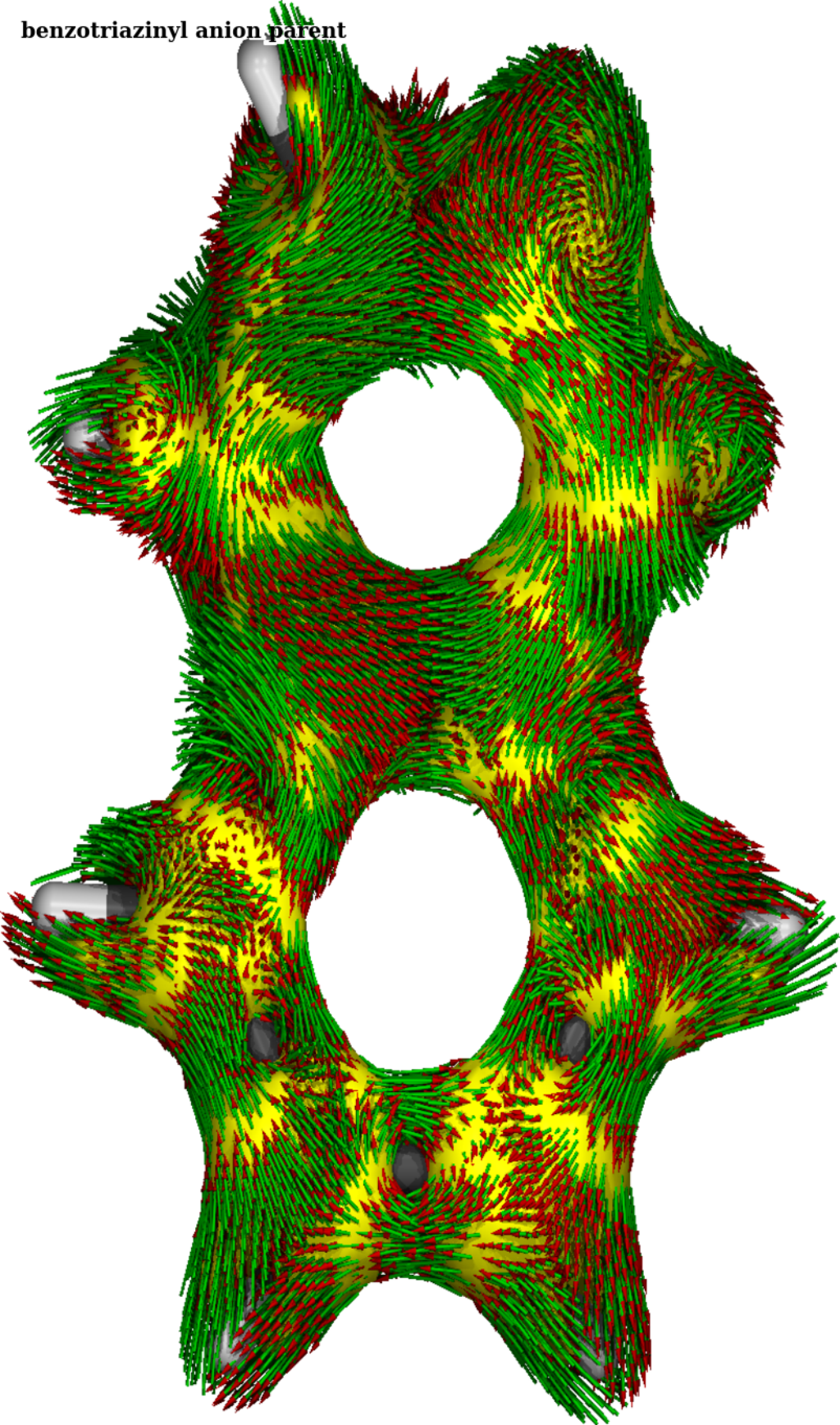

benzotriazinyl cation parent

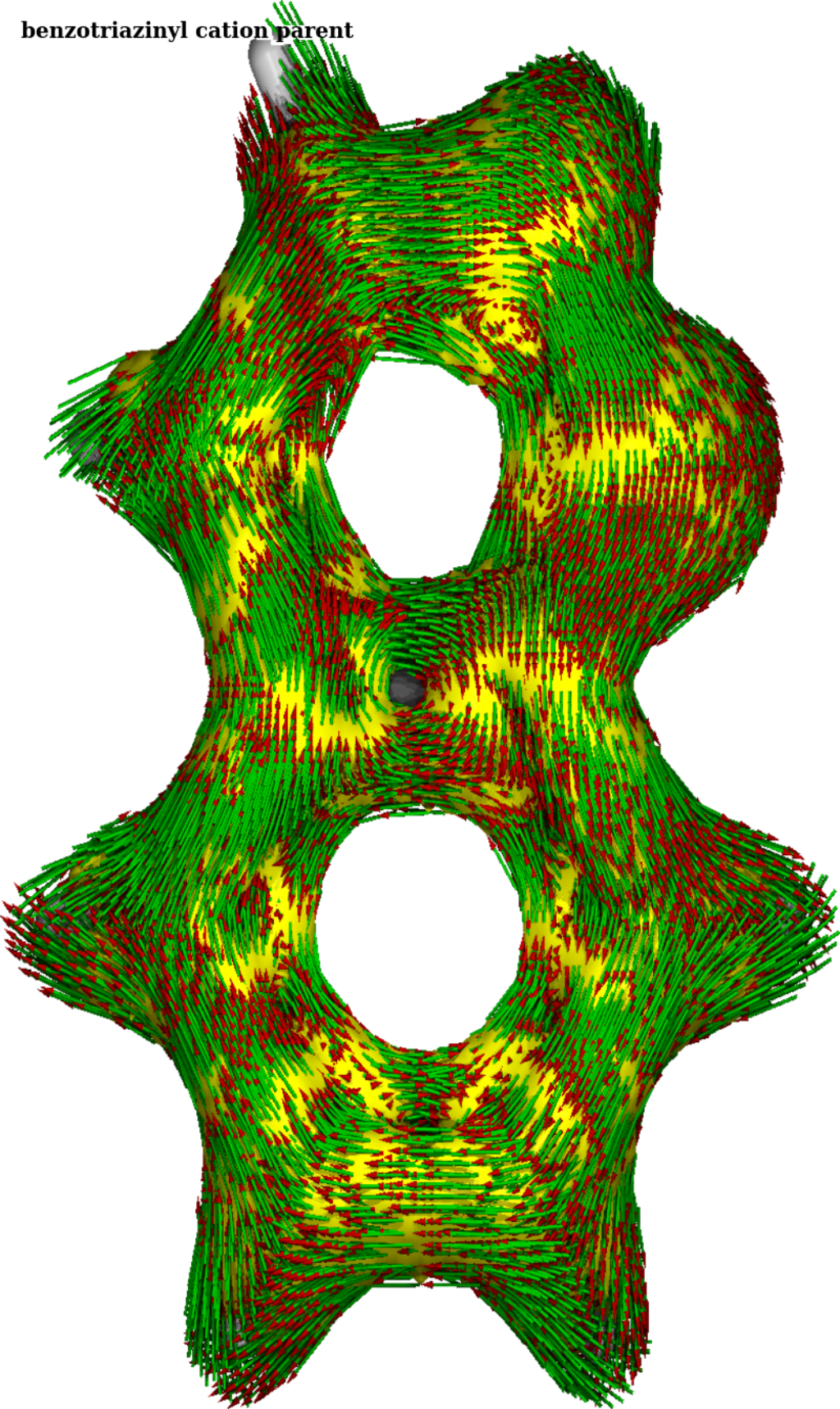

benzotriazinyl parent

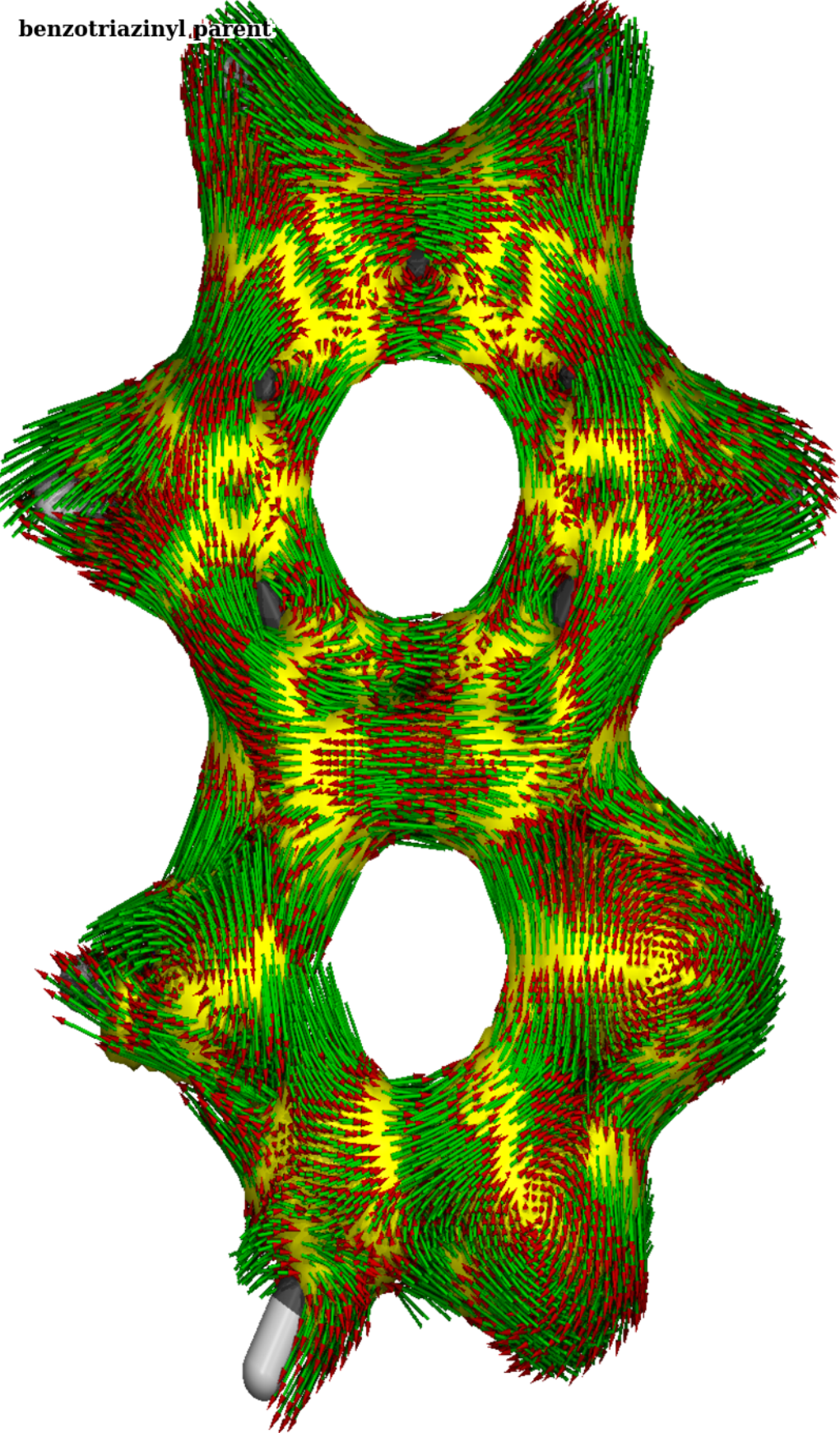

eq1 eq2 reactant

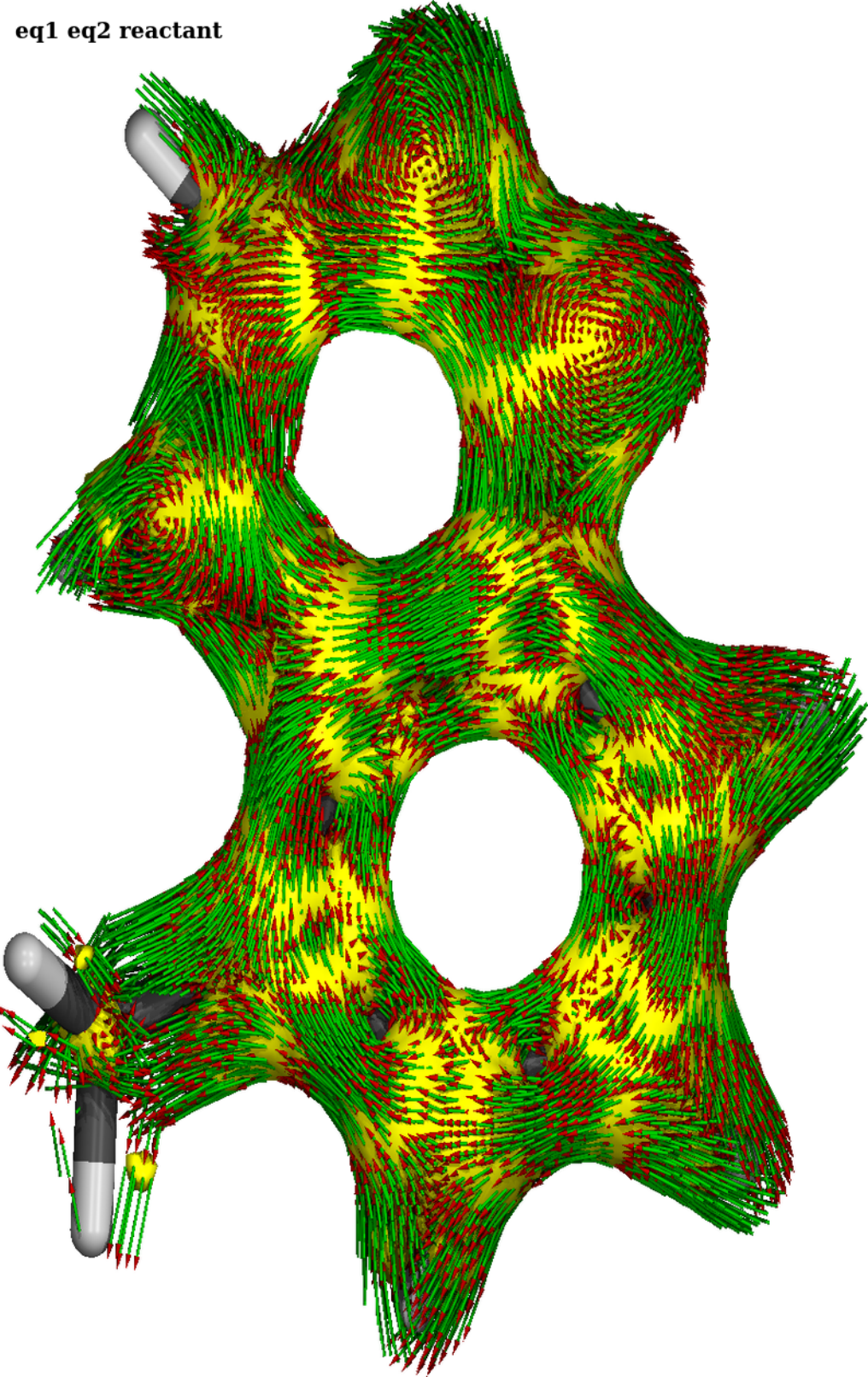

eq1 product

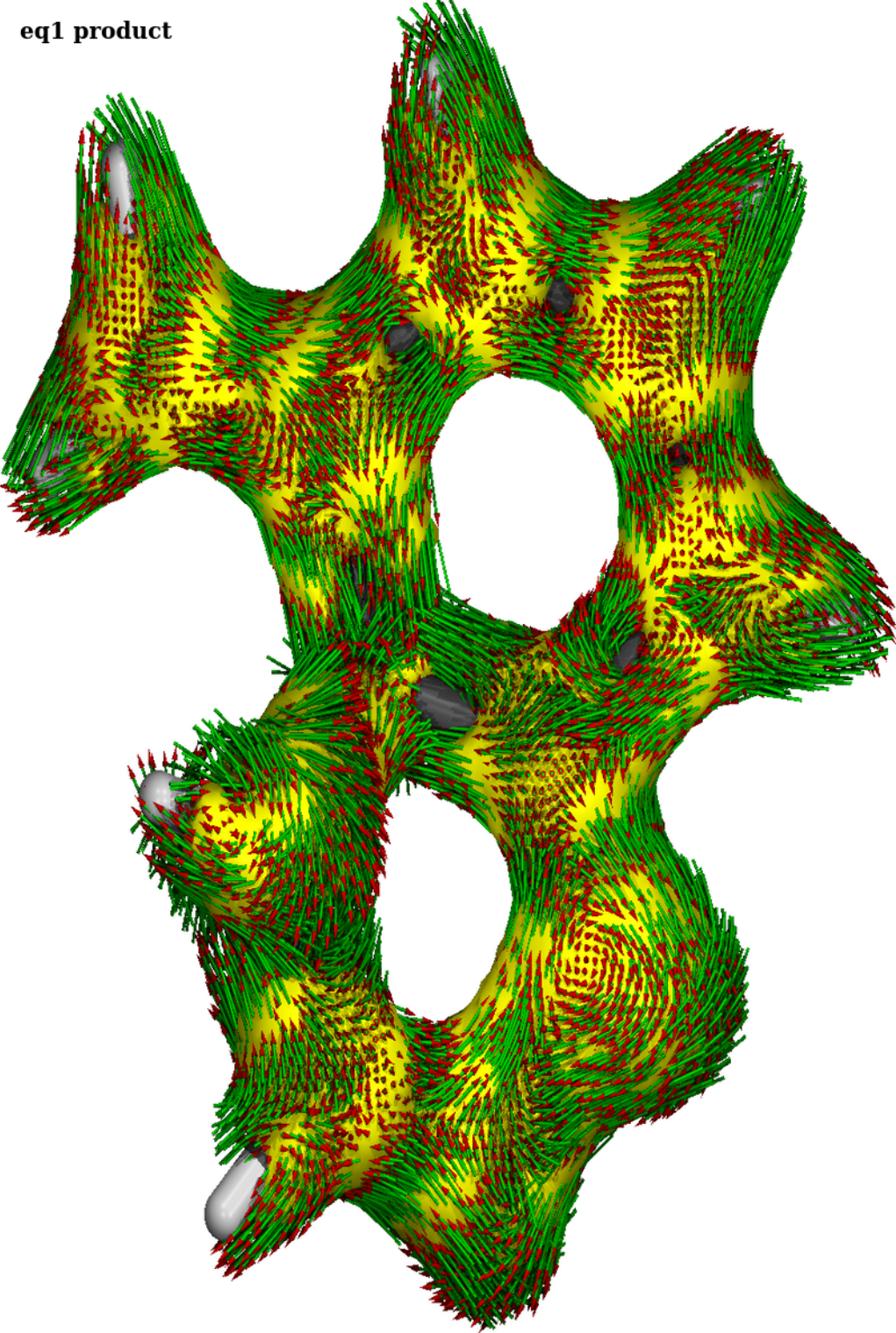

eq3 product

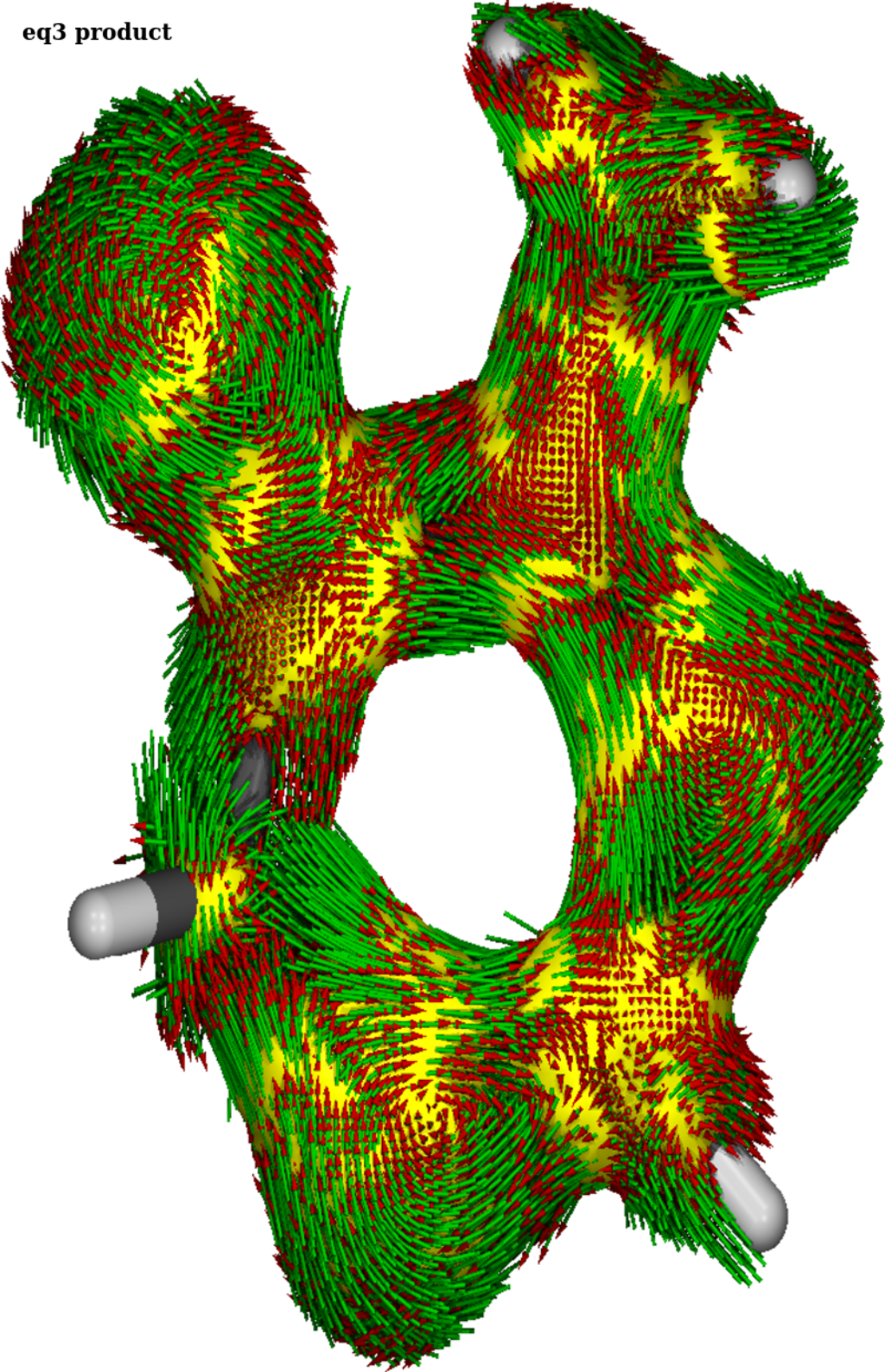

eq3 reactant

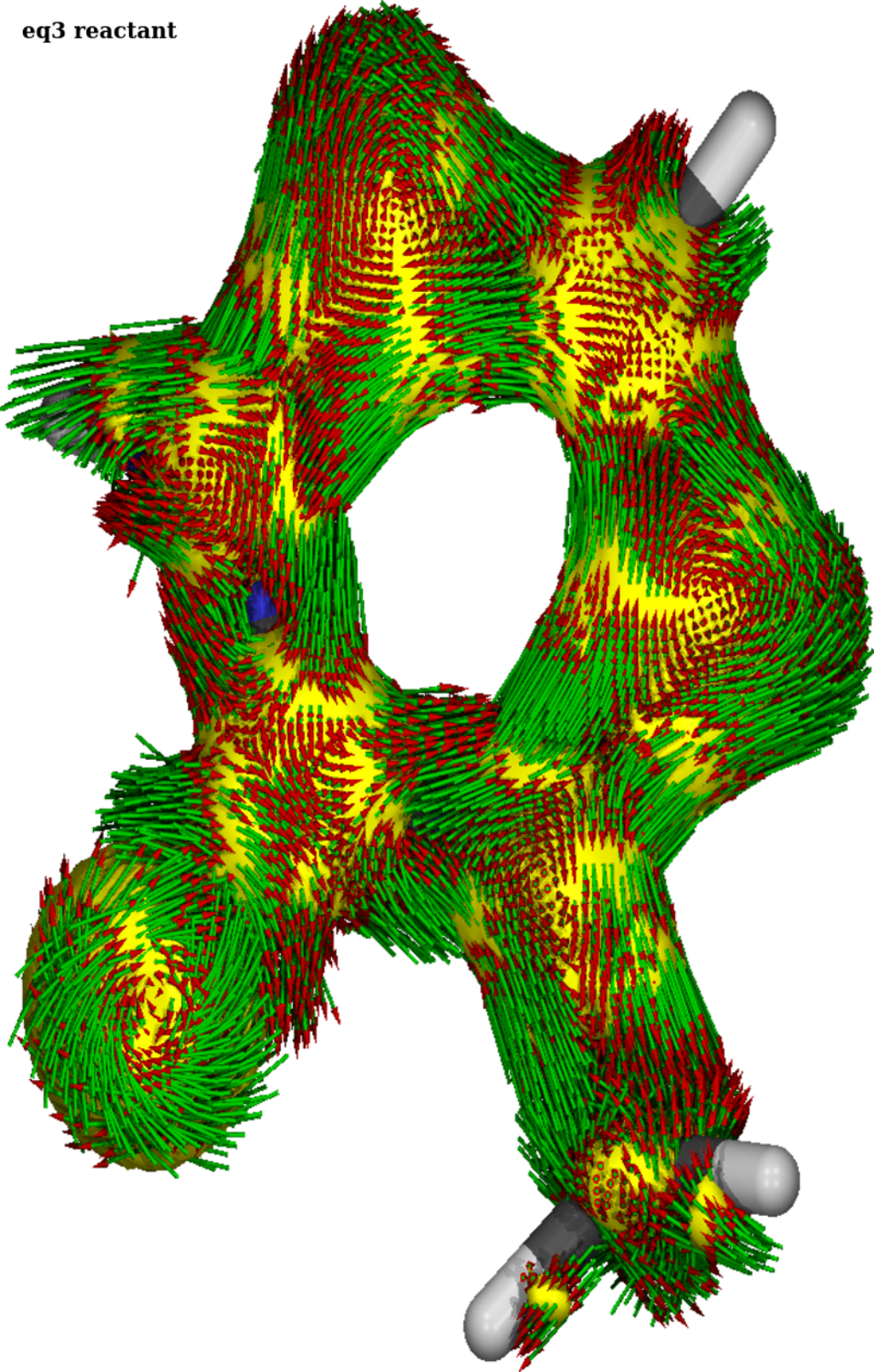

eq4 product

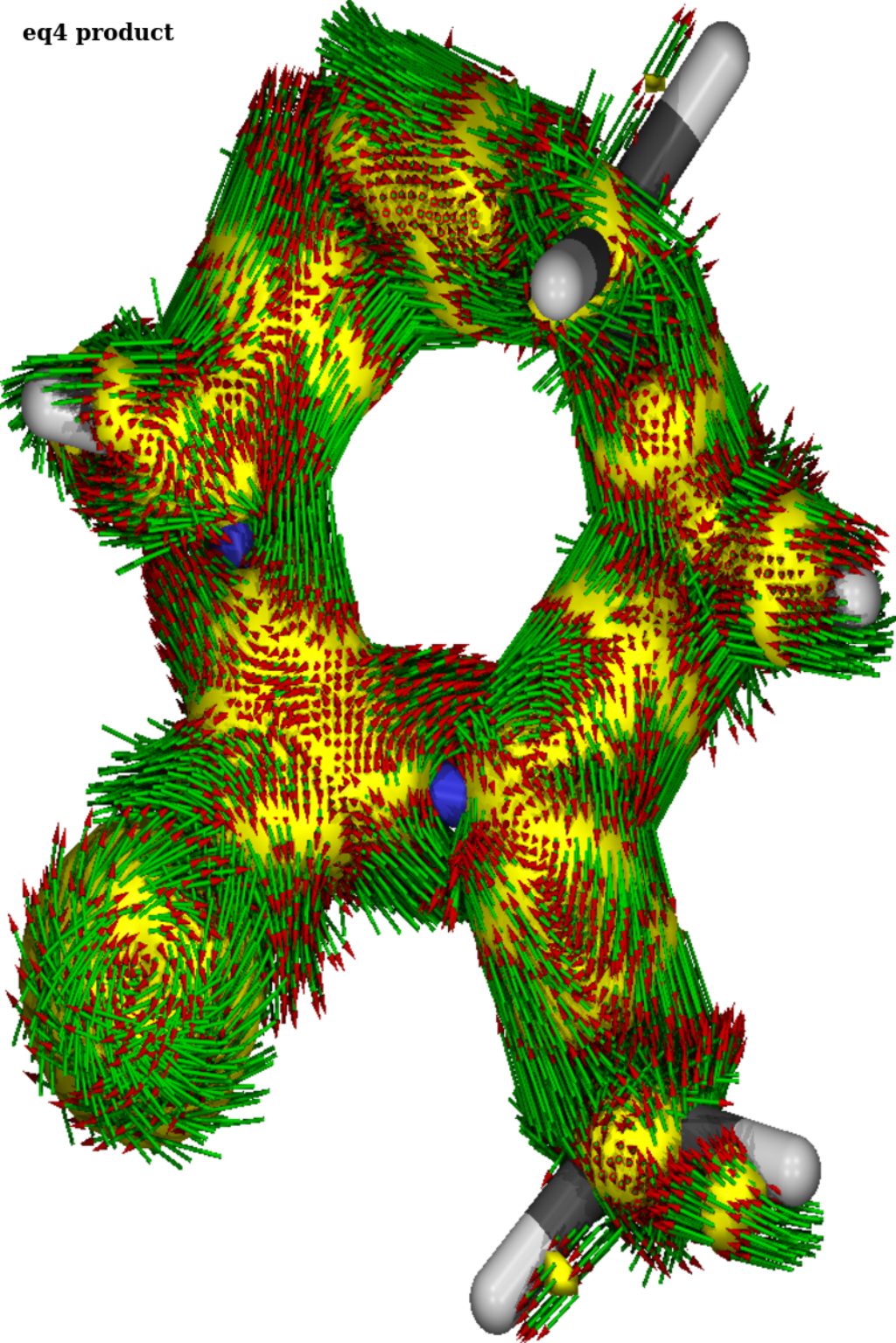

eq4 reactant

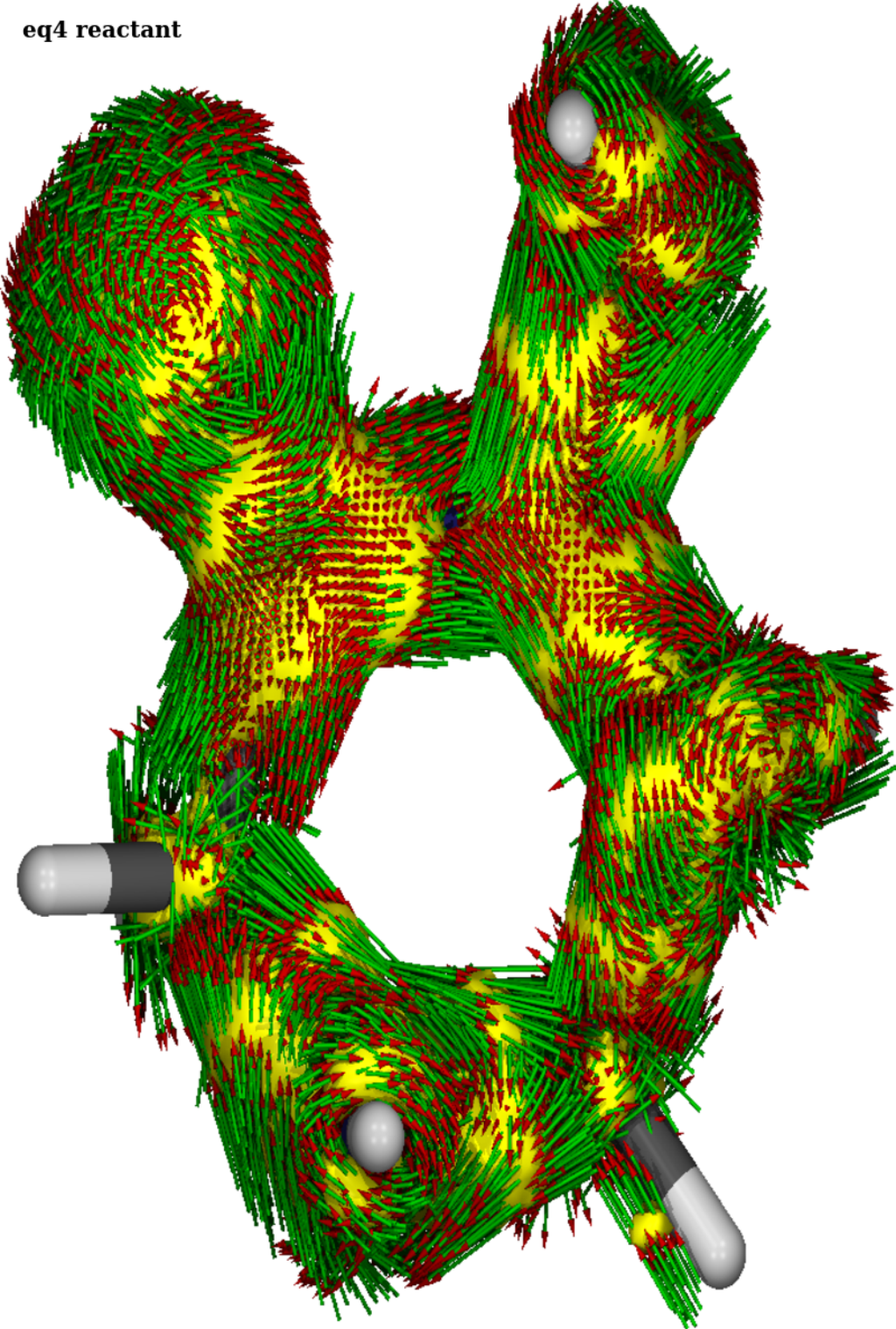

verdazinium parent

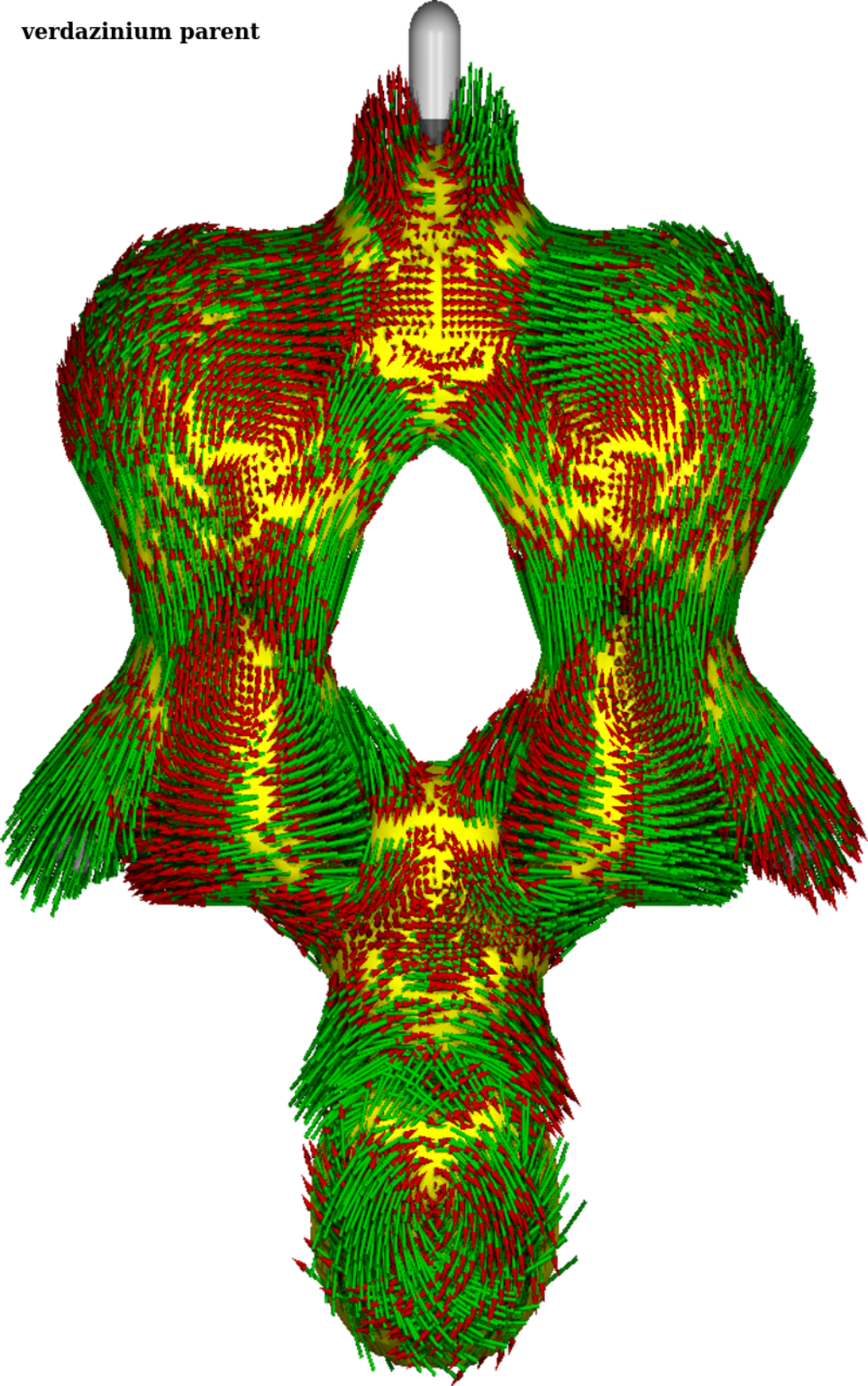

verdazyde parent

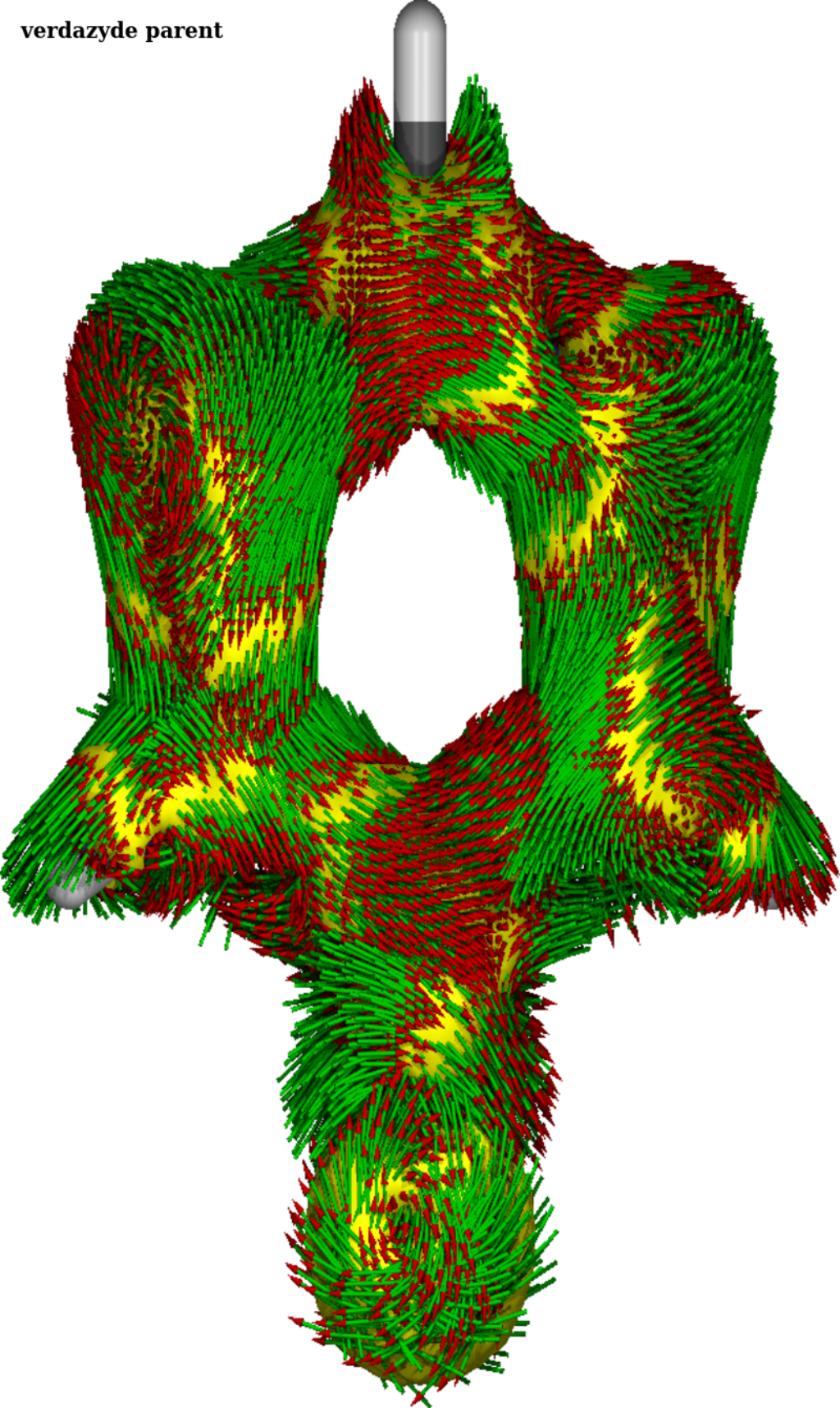

verdazyl parent

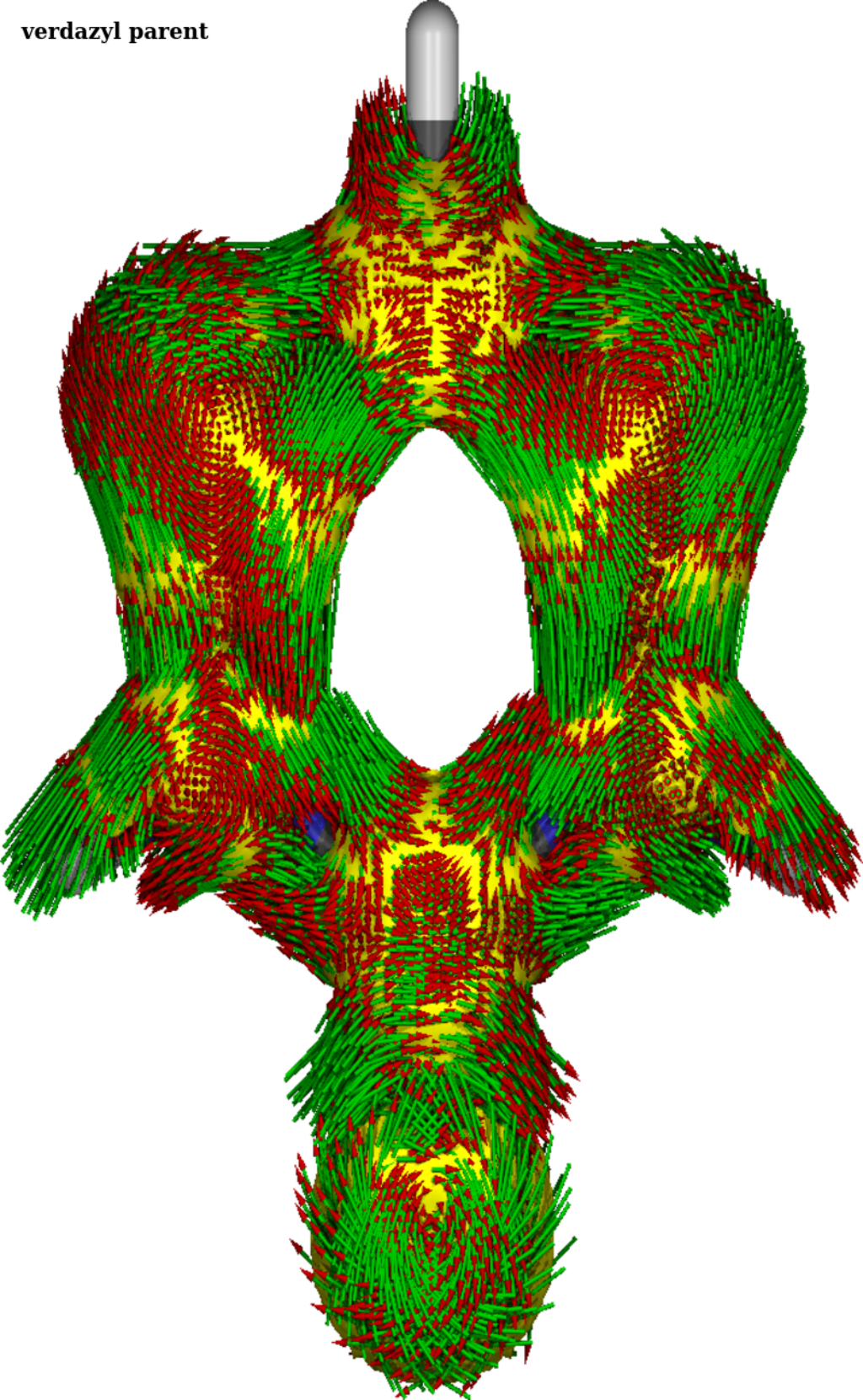

Supplement: Supplementary file 2 [file Beilstein_J_Org_Chem-22-1376-s002.pdf]
